# Design and Synthesis of Organic Molecules as Antineoplastic Agents

**DOI:** 10.3390/molecules25122808

**Published:** 2020-06-18

**Authors:** Carla Boga, Gabriele Micheletti

**Affiliations:** Department of Industrial Chemistry “Toso Montanari”, Alma Mater Studiorum - Università di Bologna, Viale del Risorgimento 4, 40136 Bologna, Italy

The fight against cancer is one of the most challenging tasks currently for lots of researchers in many fields, such as pharmaceuticals, medicine, and chemicals. In this context, synthetic organic chemistry plays a key role, representing a flexible tool that uses variations of functional groups or motifs within organic architecture; it permits the disclosure and modulation of structure and activity relationships for designing new molecules or structural hybrids once a molecular target has been identified.

A query for “anticancer” on Web of Science [1] returns more than 25,000 records just for the years of 2018 and 2019, more than half of them concerning chemical and biochemical subjects (Figure 1).

Due to the cutting-edge nature of the topic, we recognized the need to propose a Special Issue on “Design and Synthesis of Organic Molecules as Antineoplastic Agents” in the Molecules journal, with the aim of offering an appropriate opportunity to scientists to share their more relevant results within the scientific community. This volume constitutes a collection of papers for the Special Issue dedicated to the topic. 

The articles exhibit a cross-field character, focusing both synthetic aspects and biological activity of a wide variety of organic molecules, which have been often designed with the support of in silico studies. Molecular hybridization approaches have been used in several cases as a successful multi-target strategy for the design and development of novel antitumor agents.

The articles included in the collection globally show about 180 newly synthesized organic species, and more than 200 have been tested as anticancer agents.

In most cases [2,3,4,5,6,7,8,9,10,11], the structural skeleton of these molecules contain a heterocyclic scaffold, and biological investigations were supported by docking calculations to identify and predict the possible molecular targets. 

Thus, 1,2,3-triazole was used as a linker between 4-hydroxycoumarin and benzoyl-substituted arylamines to obtain novel derivatives whose biological activity was evaluated against human breast cancer MDA-MB-231 cells [2]. Novel pyrazole and benzofuropyrazole derivatives were prepared from resorcinol and tested as cell growth inhibitors in leukemia K562, lung tumor A549, and breast tumor MCF-7 cells [3]. Effects of the novel class of pyrido[3’,2′:4,5]furo(thieno)[3,2-*d*]pyrimidin-8-amines were evaluated on the methylation of DNA of murine sarcoma S-180 cells, while a docking analysis and an assessment of the anti-proliferative activity of the most active compounds were performed on both cancer (HeLa) and normal (Vero) cells [4]. Geronikaki et al. [5] report an exhaustive study on 4,5-diaryl 3(2H)furanones, ranging from synthesis to molecular docking, including a complete evaluation of the anti-inflammatory and COX-1/2 inhibitory action and the influence on cancer growth.

Both in vitro and in vivo anticancer activity for a library of 2-(het)arylpyrrolidine-1-carboxamides, synthesized by a modular approach based on the intramolecular cyclization/Mannich-type reaction of *N*-(4,4-diethoxybutyl)ureas, was tested on a M-Hela cell line [6]. Furthermore, the ability to suppress bacterial biofilm growth of some compounds bearing a benzofuroxan moiety was evaluated. A study by Mancini and Defant was based on the synthesis of new aminoquinone hybrid molecules containing covalently linked pharmacophoric units, present individually in compounds acting as inhibitors of the cancer protein targets tubulin, human topoisomerase II, and ROCK1 [7]. Docking calculation of complexes with each protein allowed the selection of some molecules to be subjected to screening on a panel of 60 human cancer cell lines. In the context of molecular hybridization, a synthesis of molecules containing azaheteroaromatics bound to azelaic acid fragments has been planned, owing to their analogous structure with some histone deacetylase inhibitors [8]. Cell lines included in the evaluation of toxicity profiles were: malignant U2OS, HT29, PC3, IGROV1, and normal human adult fibroblast HDFa cells. Biological tests on U2OS cells suggested a post-transcriptional modification of both H2/H3 and H4 histones, and an in silico investigation revealed a plausible interaction with HDAC7. Janecky et al. [9] prepared a library of 3-methylidene chroman-4-ones via the Horner–Wadsworth–Emmons methodology and made an accurate structural analysis of the products and related intermediates. Anti-proliferative activity of the compounds against leukemia and breast cancer cell lines (HL-60, NALM-6, and MCF-7) was studied, revealing that one of them promoted caspase-mediated apoptosis. Bisphenol units containing tetrahydrothiepine and dihydrothiophene derivatives as estrogen receptor α (ERα) modulators have been designed, synthesized, and investigated as therapeutic candidates for breast cancer drugs by Ohta et al. [10]. Cardiovascular activity of some nitro-substituted sulfur-containing heterocycles (a thiopyran S,S-dioxide and 3-aryl-4-nitrobenzothiochromans S,S-dioxide species) was assessed by ex-vivo studies. At the same time, molecular drug resistance (MDR) reverting effect was evaluated for selected compounds by using tumor cell lines (breast MDA-MB-453, neuroblastoma SHSY5Y, ovarian carcinoma A2780, and multidrug-resistant A2780/DX3 cells) [11]. Dihydrobetulinic-, ursolic- and oleanolic-acid derivatives have been synthesized by Spivak et al. [12], and their cytotoxicity was tested on five different human tumor cell lines (Jurkat, K562, U937, HEK, and HeLa cells) and compared with tests carried out on normal human fibroblasts. The effect of the substituent in position nine and of the functionality of methyl esterification on the biological activity of 9-hydroxystearic acid (9-HSA), an endogenous cellular lipid with anti-proliferative and selective activity against cancer cells, was evaluated [13]. The study indicated the importance, in position nine, of groups able to make hydrogen bonding with the molecular target, and the preservation of the biological activity even if the 9-HSA carboxy group is esterified.

In exploiting the effect on cancer proliferation, one may note that the reported contributions reflect a wide variety of molecular building blocks, sometimes with a natural origin. The synthesized compounds have been tested on more than 70 human cancer cell lines, besides control cells. It has been evidenced that the strategic function of docking calculations can support both synthetic design and to disclose or predict appropriate molecular targets.

The Guest Editors thank all the authors for their contributions to this Special Issue, all the reviewers for their efforts in evaluating the submitted articles, and the editorial staff of Molecules, especially the Assistant Editor of the journal Katie Zhang for gently encouraging the realization of this Special Issue.

## Figures and Tables

**Figure 1:**
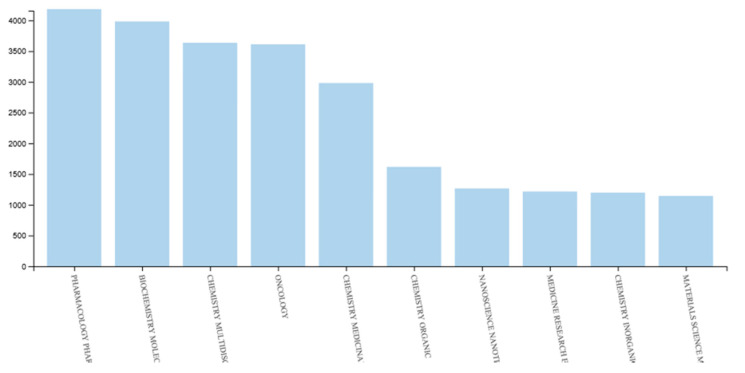
Results analysis for Web of Science query “anticancer” within the publication years of 2018 and 2019 [1].
